# Engineering and use of proteinoid polymers and nanocapsules containing agrochemicals

**DOI:** 10.1038/s41598-020-66172-w

**Published:** 2020-06-08

**Authors:** Elisheva Sasson, Ruth Van Oss Pinhasi, Shlomo Margel, Liron Klipcan

**Affiliations:** 1 The Institute of Nanotechnology and Advanced Materials, Department of Chemistry, Bar-Ilan University, Ramat-Gan, 5290002 Israel; 2 Gilat Research Center, Agricultural Research Organization, Mobile Post Negev 2, Gilat, 8531100 Israel

**Keywords:** Chemical biology, Chemical biology, Plant sciences, Plant sciences

## Abstract

To address global challenges such as population growth and climate change, introduction of new technologies and innovations in agriculture are paramount. Polymer-based formulations of agrochemicals have received much attention in recent years, and there is strong motivation to develop agrochemicals that are not harmful to the environment. Proteinoid polymers are produced by thermal step-growth polymerization of natural and unnatural amino acids. Under suitable gentle conditions, the proteinoid polymers may self-assemble to form nano-sized hollow proteinoid nanoparticles (NPs) of a relatively narrow size distribution. Agrochemical molecules may be encapsulated within these hollow proteinoid NPs, integrated in the crude proteinoid shell, or bound covalently/physically to the NP surface. In the present manuscript we prepared and characterized four model proteinoid polymers and NPs: P(KEf), P(KF), P(EWH-PLLA) and P(KWH-PLLA), where Ef denotes the unnatural herbicidal amino acid glufosinate. The NPs were fluorescently labeled and loaded with agrochemicals such as the plant hormone auxin. In addition, the NP surface was hydrophobized by covalent conjugation of dodecyl aldehyde via its surface primary amine groups. Following treatment of the plants with the different fluorescent-labeled NPs, fluorescent microscopic techniques enabled to localize the NPs and observe the accumulation in the plant’s vascular system. Next, using genetically modified plants, which express fluorescent protein and are responsive to the level of auxin, we demonstrated the possibility to deliver encapsulated agrochemicals into cells. We also illustrated that the proteinoid NPs are non-toxic to human umbilical vein endothelial cells, and apart from P(KEf) also to lettuce plants.

## Introduction

Agrochemicals are essential for sustaining modern agriculture^1^. Application of nanotechnology to agricultural production could play a fundamental role, however currently it is marginal compared with other sectors, with focus on improving agrochemical delivery and optimizing nutrient management. A recent literature meta-analysis suggests that nanoformulation improves agrochemicals delivery by about 20–30% in comparison to conventional applications—however, little is known about its influence on the environment. It is vital to develop novel products that are competitive and make agriculture more sustainable^2^.

Proteinoids – are amino acid-based polymers which are synthesized by thermal step-growth polymerization mechanism. Proteinoids were discovered by Fox *et al*. while imitating primordial conditions leading to the origins of life^3–5^. Under certain conditions, proteinoids may self-assemble to form hollow nanoparticles (NPs) by a process driven by the hydrophilic/hydrophobic balance within the proteinoid backbone^6^; in order to minimize contact with the aqueous media, the hydrophobic residues form a hydrophobic core, whereas hydrogen bonds are formed with hydrophilic residues on the NP outer shell. When self-assembly is performed in the presence of suitable molecules, proteinoid nano/micro-capsules can entrap these molecules within the particle core. Hence, proteinoid NPs could be utilized in many fields such as drug delivery, disease diagnosis, and coatings^6–14^. Previous studies by our group presented proteinoid NPs that entrap fluorescent dyes for imaging^15^, vitamin A for cosmetics^10^, and drug molecules^9,15^ such as doxorubicin for cancer therapy^4–6,8^. Furthermore, in our previous publications, we illustrated that the density of dry proteinoid capsules ranges from 0.001 to 0.014 g/mL suggesting that hollow particles are formed^16^, as described by Fox *et al*.^17^. In addition, increasing the concentration of the encapsulated compound, leads accordingly to an increase in the diameter of the particles^18,19^, similar to blowing an empty hollow ball with air.

Here, a new idea is demonstrated whereby various amino acids including currently used herbicides and potential biostimulants undergo random polymerization by a thermal step-growth mechanism to form herbicidal/biostimulant proteinoid NPs with enhanced stability and/or delivery via the roots and/or leaves. These agrochemical-containing proteinoids are protein-like, however they have no secondary and tertiary structure. They are non-toxic, non-immunogenic and biodegradable^6–10,20^ and due to the presence of amide and ester groups, may enable controlled release of the desired molecules into the weed environment. In addition, as the proteinoid NPs exhibit various functional groups on the surface, e.g. primary amines and carboxylates, they may be used for covalent conjugation of various functional compounds. Here, we covalently conjugated dodecyl aldehyde (DA) to the proteinoid NP surface to increase its hydrophobicity; alternatively, the fluorescent dye Cyanine3 NHS ester (Cy3 NHS) was conjugated to allow tracking the movement of the NPs within the plant.

## Materials and methods

The following analytical-grade chemicals were purchased from commercial sources and used without further purification: L-glutamic acid (E), L-phenylalanine (F), L-histidine (H), L-lysine (K), L-tryptophan (W), glufosinate (Ef), poly(L-lactic acid) (PLLA, Mw of 2 kDa), dodecyl aldehyde (DA), sodium cyanoborohydride, poly(ethylene glycol) NHS ester, Cyanine3 NHS ester (Cy3 NHS), auxin (sodium salt), doxorubicin, human serum albumin (HSA), Triton-x-100, bovine plasma fibrinogen, Murashige and Skoog (MS) and plant agar from Sigma (Rehovot, Israel); phosphate buffered saline (PBS), minimum essential medium Eagle’s supplement (MEM), fetal bovine serum (FBS), glutamine, penicillin, streptomycin, sodium 3´-[1-(phenylaminocarbonyl)-3,4-tetrazolium]-bis(4-methoxy-6-nitro) benzene sulfonic acid hydrate (XTT) and mycoplasma detection kits from Biological Industries (Bet Haemek, Israel); human umbilical vein endothelial cells (HUVEC) and their culture medium EGM-2 from Lonza Israel; Water was purified by passing deionized water through an Elgastat Spectrum reverse osmosis system (Elga Ltd., High Wycombe, UK).

### Preparation of proteinoid polymers

Proteinoid synthesis followed our previous publications^6–9^; lysine or glutamate (or aspartate) have a major role in this process by initiation the polymerization^6,21,22^. Thus, all proteinoids used in these studies contain glutamate and/or lysine in their main chains. Briefly, different combinations of the natural and non-natural amino acids: L-glu or L-lys, L-phe, L-his, L-trp and glufosinate (Ef, Fig. S1c) were first mixed giving, in absence or presence of PLLA, a total monomer weight of 5 g. Different weight ratios were analyzed, as specified in Table 1. Each mixture was heated in a mantle, under nitrogen conditions, to 180 °C. The mixture was mechanically stirred at 150 rpm for 45 min to yield while at 180 °C until a brown glassy mass was formed and was allowed to cool to room temperature. Water (15 mL) was added to the crude product, and the mixture was stirred overnight. The non-soluble part was removed by filtration and weighed, while the water-soluble part was dialyzed intensely against distilled water using a cellulose membrane (500–1000 Da MWCO). The contents of the dialysis membrane were lyophilized and a yellow-white proteinoid polymer powder was obtained.

**Table 1:** Proteinoid amino acid and PLLA content.

Proteinoid polymer	Amino acid and PLLA content (g)						
	L-Lys	L-Glu	L-Phe	L-His	L-Trp	Ef	PLLA
P(KEf)	3.5					1.5	
P(KF)	2.5		2.5				
P(EWH-PLLA)		1.5		1.5	1.5		0.5
P(KWH-PLLA)	1.5			1.5	1.5		0.5

### Characterization of the different proteinoid polymers

Gel permeation chromatography (GPC) was used in order to analyze the molecular weight and polydispersity index of the proteinoids, as described earlier^8^. GPC spectra were obtained with a Waters Spectra Series P100 isocratic high performance liquid chromatography (HPLC) pump with UV/vis detector, ERMA ERC-7510 refractive index detector, and a Rheodyne (Coatati, CA) injection valve with a 20 µL loop (Waters, MA). Samples were eluted with super-pure HPLC water through a linear BioSep SEC-s-3000 column (Phenomenex) at 1 mL/min flow rate. Poly(ethylene glycol) standards (Polymer Standards Service, Silver Spring, MD, USA) with molecular weights between 100–450,000 Da, HSA (67 kDa), and bovine plasma fibrinogen (340 kDa) were used to determine the molecular weights using Clarity chromatography software. A PE 343 polarimeter (PerkinElmer, MA, USA) was used to determine the optical activity of the proteinoids. All measurements were performed at 25 °C in water at 589 nm.

### Preparation of proteinoid NPs

Hollow proteinoid NPs were prepared by self-assembly. In brief, 100 mg of dried proteinoid were added to 10 mL of a 10^−5^ N NaCl aqueous solution. The proteinoid polymer mixture was heated to 80 °C until dissolution. Upon removal of the heating, the mixture underwent slow cooling to room temperature, and proteinoid NPs were formed by a self-assembly process. The obtained capsules were dialyzed with a cellulose membrane (500–1000 Da MWCO) against distilled water in order to eliminate excess reagents^7,15,16,18,19,23,24^.

### Synthesis of fluorescent proteinoid NPs

**Figure 1:**
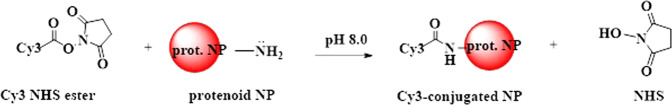
Conjugation of Cy3 NHS ester to the proteinoid NPs.

Fluorescent proteinoid NPs were prepared in order to track the movement of the particles in plants. Cy3 fluorescent dye labeling is the most suitable for plants, as its fluorescence wavelength is different than chloroplasts^25^. Cy3-NHS ester was conjugated to the primary amine groups on the surface of proteinoid NPs (Fig. 1). In brief, Cy3 NHS ester (0.5 mg) dissolved in 50 µL of anhydrous DMSO was added to 10 mL of aqueous NP dispersion (10 mg/mL in NaCl, 10^−5^ M) and mixed at room temperature for 1 h. The obtained fluorescent dye-conjugated hollow NPs were rinsed from excess reagents by extensive dialysis (500–1000 Da MWCO) against distilled water.

### Encapsulation of auxin within proteinoid NPs

To 10 mL of a 10^−5^ N NaCl solution, 10 mg P(EWH-PLLA) proteinoid polymer and 0.5 mg auxin, sodium salt, 5% w/w, (Fig. S1a) were added. The mixture was heated to 80 °C until dissolution. P(EWH-PLLA) NPs containing entrapped auxin were then prepared by removal of the heating and slow cooling to room temperature, followed by extensive dialysis against distilled water to wash off excess reagents. A similar process was done for encapsulation of auxin in P(KWH-PLLA) NPs.

### Conjugation of dodecyl aldehyde (DA) to the proteinoid NP surface

**Figure 2:**
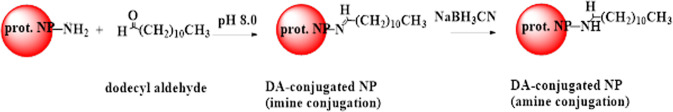
Conjugation of DA to proteinoid NPs showing intermediate imine conjugation.

Dodecyl aldehyde (DA) was covalently conjugated to the P(KEf) NPs by formation of Schiff base bonds via the interaction of the aldehyde groups of DA with primary amine groups on the NP surface (Fig. 2). Briefly, the pH of the aqueous proteinoid NP dispersion (100 mg/10 mL NaCl, 10^−5^ M) was increased to 8.0 by gradual addition of 0.1 M NaOH. Thereafter, 1 and 10 mg of DA (1 and 10% relative to the proteinoid NP weight) were dissolved in 100 µL DMSO and mixed at room temperature for 12 h. Reduction of the formed imine bonds to stable amine bonds was done by adding 10 mg sodium cyanoborohydride to the DA-conjugated NPs dispersion, followed by mixing at room temperature for additional two hours. The obtained DA-conjugated NPs were washed from excess reagents by extensive dialysis (500–1000 Da MWCO) against distilled water.

### Proteinoid NPs characterization

#### Size and size distribution

The dry diameters and diameter distributions of the proteinoid NPs were measured at room temperature with a high-resolution scanning electron microscope (HR-SEM). Images were obtained with a JEOL, JSM-840 Model (Japan). A drop of dilute particle dispersion was spread on a glass surface and was allowed to dry at room temperature. The dried samples were coated with iridium before viewing under a HR-SEM in vacuum. More than 200 particles were measured with image analysis software (Analysis Auto, Soft Imaging System GmbH, Germany) to determine the dry diameter and distribution of the NPs.

The hydrodynamic diameters and diameter distributions of the proteinoid NPs dispersed in aqueous media were measured at room temperature by a Vasco 2 DLS analyzer particle sizing system (Cordouan Technologies SAS, France).

#### FTIR

FTIR measurements of the proteinoid NPs were obtained by the attenuated total reflectance (ATR) technique using a Bruker Alpha-FTIR QuickSnape sampling module equipped with a platinum ATR diamond module.

#### Fluorescence characterization

The fluorescence of Cy3-conjugated proteinoid NPs was compared with free Cy3. Samples were diluted with pure water to avoid inner filter effect (100 µl NPs in 900 µl H_2_O). The dilution ratio was determined after measurements at successive dilution ratios in order to limit overlapping signals. Samples were measured with a multiplate reader Synergy 4 (BioTek instruments, Winooski, VT, US). The scanning range was 400–650 and 550–650 nm for excitation and emission respectively. Excitation and emission slits were fixed at 5 nm, and λ*ex* and λ*em* were set at 540 and 650 nm, respectively. All spectra were normalized.

#### Fluorescence photostability of the proteinoid NPs

An aqueous solution of free Cy3 was prepared in order to provide the same fluorescence intensity as the corresponding Cy3-conjugated NPs aqueous dispersion. The fluorescence intensities of the free and conjugated Cy3 were measured at excitation and emission wavelengths *λex* and *λem* set to 540 and 650 nm, respectively. Samples were illuminated continuously with a xenon lamp, and the fluorescence intensity of Cy3 was measured over 30 min by a Synergy fluorescence spectrophotometer (Agilent Technologies Inc.) and normalized to allow comparison.

#### Encapsulated auxin determination by HPLC

The encapsulated auxin concentration was determined using HPLC analysis (Spectra System HPLC equipped with UV/vis detector, Thermo Scientific, USA). The analysis was performed using a reverse phase C18 column (75 mm × 4.6 mm, Phenomenex, USA) with water and acetonitrile mobile phase containing 0.01% trifluoroacetic acid (added from concentrated aqueous solution) at 1 mL/min flow rate. Calibration standard solutions of auxin in the range of 10–600 µM were prepared by dilution in acetonitrile. Prior to injection, samples composed of auxin-containing proteinoid NPs were diluted in acetonitrile and sonicated for 4 min in an ice-water bath. Following sonication, the proteinoid NPs disassembled and eluted the auxin. The injection volume was set to 50 µL, and the concentration of encapsulated auxin was calculated using the calibration curve.

#### Proteinoid NPs stability

Aqueous dispersions of non-modified and modified proteinoid NPs (1 mg/mL Cy3 and DA-conjugated as well as auxin-containing NPs) were stored for six months at 4 °C. The size and size distributions of the dispersions were analyzed by HR-SEM. In addition, the NP stability was evaluated by HR-SEM following freeze-drying and re-dispersion in aqueous phase at the original concentration.

#### *In vitro* cytotoxicity

*In vitro* cytotoxicity was tested using human umbilical vein endothelial cells (HUVEC). An XTT assay was performed to determine the viability of the HUVEC cells after proteinoid NPs treatment. The assay is based on the ability of the mitochondrial succinate-terazolium reductase system to convert the yellow tetrazolium salt XTT (sodium3´-[1-(phenylaminocarbonyl)-3,4-tetrazolium]-bis(4-methoxy-6-nitro) benzene sulfonic acid hydrate) to orange formazan dye^26^. The test provides the cells viability level (mitochondrial level activity) after toxic exposure. Cells were seeded in a 96-well plate at a density of 10^4^ cells/well in 100 μL culture medium and grown in a humidified 5% CO_2_ atmosphere at 37 °C. After 48 h at 37 °C, different volumes of NPs dispersed in PBS were added to the cells, yielding final concentration of 1 mg/mL per well. After incubation for 48 h at 37 °C, 50 μL of XTT solution were added to each well according to the kit manufacturer’s instructions. Absorbance was read at 488 nm. Cell viability was determined using the formula in the manufacturer’s protocol. The reference wavelength was 620 nm.

### Proteinoid NPs in plants

#### Seed germination assay

Proteinoid NPs were tested on lettuce (*Lactuca sativa*) for possible effects on seed germination. A sample was collected from previously used soil from different experiments performed in the lab. Growth media, potting mix (10 g containing peat, composted bark and sand as amended with 30 mL each of aqueous dispersion containing P(KF), P(KEf), P(EWH-PLLA) and P(KWH-PLLA) NPs at various concentrations to yield a final concentration of 0.25 mg/mL in each Petri dish. Soil amended with 30 mL of untreated purified water served as control so that each dish was moistened with an equal volume of solution before an experiment was conducted. Lettuce seeds were sown after the proteinoid NP aqueous dispersion was mixed with the soil media. All dishes were closed with lids after seeding and placed in a controlled growth room at 25 °C at 16:8 h light/dark cycle under constant light (200 micro-Einstein) until experiment termination. After five days of growth, root lengths of seedlings were measured.

#### Root accumulation

Accumulation of Cy3-conjugated P(KEf) NPs in the roots was performed using tomato (*Solanum lycopersicum* M82) plants. Seedlings were grown for four weeks in polyethylene containers filled with commercially available growth media in a growth room (25 °C, 16:8 h light-dark cycle). Then, the Cy3-conjugated proteinoid NPs aqueous dispersions (10 mg/mL) were applied to the media. Roots of tomato were washed with distilled water before microscopic analysis, and cut vertically or perpendicular to the growth axis to visualize the inner anatomical structure. Accumulation of NPs in different plant tissues was photographed using a Nikon SMZ-25-Fluorescence Stereoscope equipped with high resolution DIS-Ri2 color camera and running NIS-Elements-BR software after 24 h post media application.

#### Leaf movement

Lettuce plants were grown for one week in polyethylene containers filled with commercially available growth media in a controlled environment greenhouse (approx. 30/20 °C during day/night). Then, aqueous dispersions (10 mg/mL) of Cy3-conjugated P(KEf) NPs were applied on the top leaf. Accumulation of the NPs in different plant tissues was observed using a fluorescent stereoscope at 72 h post foliar application. Leaves of tomato were washed with distilled water prior to analysis.

#### DR5:YFP transgenic background tomatoes

Transgenic tomatoes DR5:YFP (*Solanum lycopersicum* M82) lines were previously described by Pattison *et al*.^27^. Seedlings were grown for four weeks in polyethylene containers filled with commercially available growth media in a growth room (25 °C, 16:8 h light-dark cycle). The 30 day old DR5-YFP seedlings were sprayed (for foliar treatment) or treated (for roots treatment) with 350 µL of non-encapsulated and auxin-encapsulated P(EWH-PLLA) and P(KWH-PLLA) aqueous dispersions (10 mg/mL) of NPs containing 4.6 and 4.2 weight %, respectively, of encapsulated auxin. After 48 h incubation in a growth chamber, expression of yellow fluorescent protein (YFP) was analyzed by fluorescent binocular and compared to non-treated DR5:YFP plants.

#### Phytotoxicity test – foliar application

Lettuce plants were grown for one week in polyethylene containers filled with commercially available growth media. Foliar treatments consisted of control, non-conjugated, and DA-conjugated non-fluorescent and fluorescent P(KEf) NPs. Plants received one treatment, and results were analyzed visually and by fluorescent binocular after ten days.

### Data analysis

Data was analyzed for statistical significance among means by using the Student’s *t*-test or one-way ANOVA followed by comparison of means using the Tukey–Kramer HSD test (JMP software, http://www.jmp.com). Significant difference was considered when *P* < 0.05.

## Results and discussion

### Characterization of proteinoid polymers

Proteinoid polymers were prepared by thermal bulk step-growth polymerization of different standard and non-standard amino acids including the widely-used herbicide glufosinate (Ef, Fig. S1c) both with and without PLLA. Table 1 shows the amino acid content used for the four proteinoid polymers employed in the present study: P(KF), P(KEf), P(EWH-PLLA) and P(KWH-PLLA). The proteinoids possess a protein-like primary structure that grants them biocompatibility, non-toxicity, and immuno-safety^4–9,13,14,28–30^, they are random polymers, with no secondary or tertiary structure, that can be branched or cross-linked by functional groups such as amine and carboxyl belonging to lysine and glutamic acid, respectively. This cross-linked part was removed from the linear (soluble) part by filtration.

The crosslinked part was between 5–20%, depending on the proteinoid composition. The obtained soluble proteinoid polymers were characterized in terms of molecular weight, polydispersity, and optical activity, as shown in Table 2. The synthesized proteinoids were of relatively high molecular weight, in the range of 88–356 kDa with low polydispersity index (PDI) in the range of 1.0–1.05. This surprising result for step-growth polymerization is in accord with our previous results, indicating that thermal condensation polymerization of amino acids, prepared according to our publications, provides relatively long polymer chains with low PDI^6–10^. Table 2 also shows that the molecular weight of the obtained proteinoids is highly dependent on its composition. For example, substitution of F for Ef resulted in over 1.3-fold increase (130 *vs*. 95 kDa, respectively), while substitution of E in for K (with PLLA) resulted in about 4-fold increase (356 *vs*. 88 kDa). Table 2 also shows that all proteinoids exhibit optical activity, although the monomers are known to racemize during thermal polymerization. This optical activity may be useful in the future for various biomedical applications.

**Table 2:** Molecular weight, polydispersity and optical activity of the proteinoid polymers^a^.

Proteinoid polymers^a^	Mw (kDa)	Mn (kDa)	Mp (kDa)^b^	PDI	Opt. activ. [α]_D_^25 °C^ (°)
P(KEf)	130	128	130	1.01	+26.5
P(KF)	95	87	100	1.05	−5
P(EWH-PLLA)	88	86	82	1.02	−8
P(KWH-PLLA)	356	351	322	1.01	10

^a^Molecular weights were measured by GPC, Mp is the molecular weight at the peak, PDI is the polydispersity index given by Mw/Mn, specific optical rotation was measured in H_2_O at 25 °C (c = 1). Each experiment was performed four times. The standard deviation was 0.5–2%.

### Characterization of proteinoid NPs

#### Proteinoid NP size and size distribution

**Figure 3:**
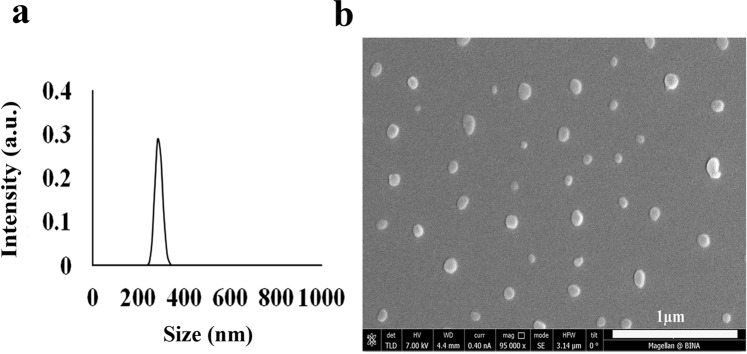
Hydrodynamic size histogram (**a**) and HR-SEM image (**b**) of P(KEf) NPs.

Table 3 summarizes the dry (HR-SEM measurements) and hydrodynamic (light scattering measurements) diameters and diameter distributions of the proteinoid NPs prepared in the present study. The dry diameter is almost always lower than the hydrodynamic diameter, as light scattering also takes into account the hydrated water layers adsorbed onto the particle surface, as well as the water molecules entrapped within the proteinoid NPs. Non-agglomerated elliptic NPS were produced with relatively narrow hydrodynamic diameters of 284 ± 25, 155 ± 11, 307 ± 12, 205 ± 12, and dry size diameters of: 76 ± 19, 59 ± 14, 135 ± 24 and 85 ± 10 nm for P(KEf), P(KF), P(EWH-PLLA) and P(KEWH-PLLA), respectively. For illustration, Fig. 3 exhibits the typical HR-SEM image and hydrodynamic size histogram of the P(KEf) NPs. Similar images were observed for the other proteinoid NPs. Table 3 clearly shows that the average diameter of these NPs depends on their chemical composition – replacing K by E produced nearly 60% increase, while substituting F by Ef resulted in nearly 30% increase. It is noticeable that the NPs with lower diameter have higher permeability to the plants, and thereby improved activity^31^.

**Table 3:** Proteinoid NP size and size distribution^a^.

Proteinoid NPs	Hydrodynamic diameter (nm)^b^	Dry diameter (nm)^b^
P(KEf)	284 ± 25	76 ± 19
P(KF)	155 ± 11	59 ± 14
P(EWH-PLLA)	307 ± 12	135 ± 24
P(KWH-PLLA)	205 ± 12	85 ± 10

^a^Particle diameters were measured by light scattering measurement and HR-SEM.

^b^Errors indicate the standard deviation (*n *> 200).

#### FTIR

FTIR was used as an additional method to characterize the particles. FTIR spectra of the proteinoid NPs are illustrated in Fig. S2. Characteristic peaks are apparent at 1134 and 1030 cm^−1^ corresponding to P = O and P = OH stretching bands, 3360 and 2930 cm^−l^ typical of NH stretching, 2930 and 2869 cm^−1^ corresponding to the CH alkyl stretching band, amide CO stretching at 1514 − 1654, amide NH bending band at 1449, and CO bending at 500 − 700 cm^−1^.

### Characterization of fluorescent Cy3-conjugated NPs

**Figure 4:**
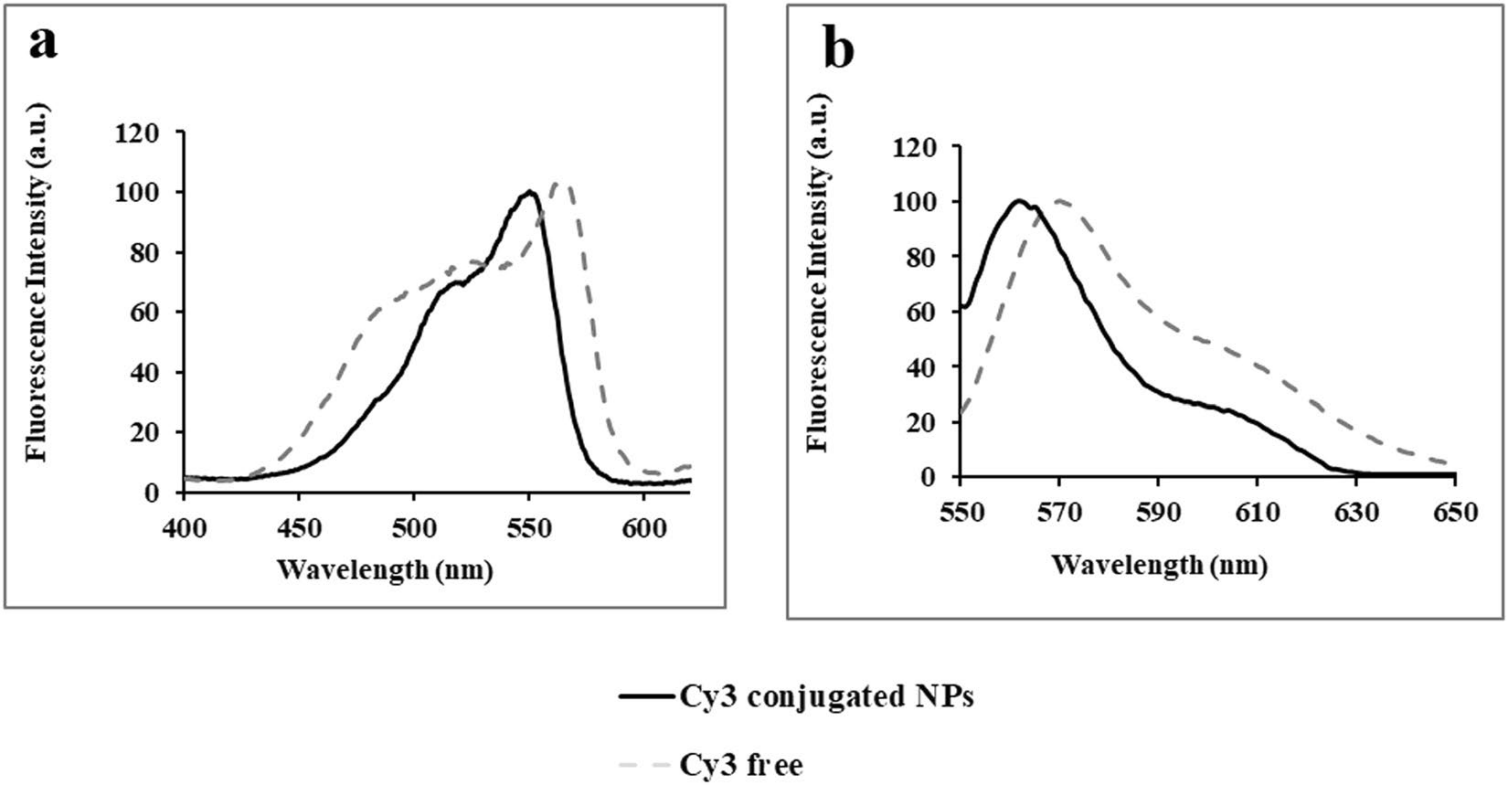
Normalized **(a)** excitation **(b)** and emission spectra of free Cy3 dye (dashed gray line) and Cy3-conjugated P(KEf) NPs (black line).

Cy3-conjugated proteinoid NPs were synthesized by reacting the primary amine groups on the surface of the proteinoid NPs with Cy3 NHS ester, as shown in Fig. 1. This type of reaction is common for covalent binding of desired ligands, e.g. antibodies, polyethylene glycol (PEG) of different lengths, drugs and dyes to nano/micro-particles containing surface primary amino groups^32–35^. Excitation and emission spectra of free Cy3 and Cy3-conjugated P(KEf) NPs are exhibited in Fig. 4. The maximum excitation intensity of free and conjugated Cy3 appears at 568 and 550 nm, respectively, while the maximum emission intensity is slightly upfield at 570 and 561 nm, respectively. This blue shift may be attributed to the change in dipole moment due to covalent binding of the Cy3 dye^36^.

**Figure 5:**
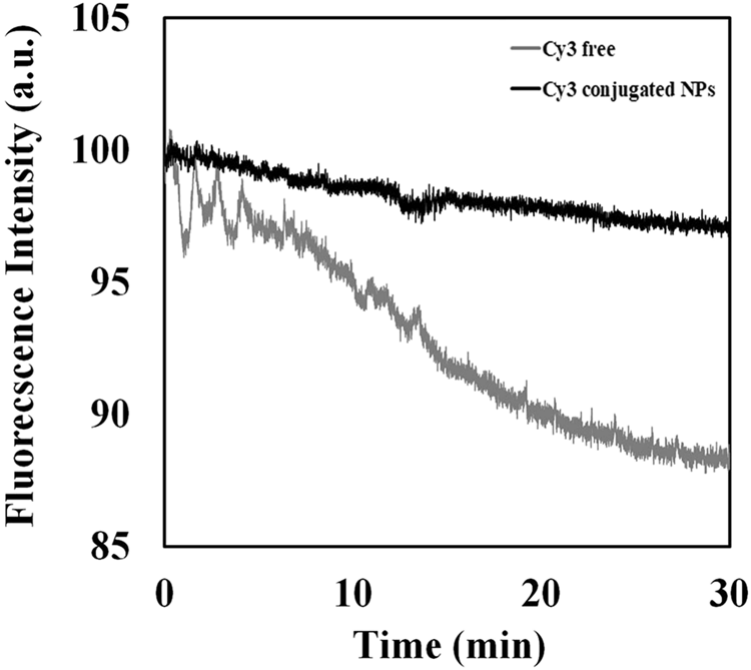
Reduction in photostability of free (gray) *vs*. conjugated (black) dye.

Photostability experiments were performed by illuminating the samples at 554 nm, and fluorescence intensities were recorded over 30 minutes. Figure 5 exhibits improved photo-stabilization of the conjugated fluorophore compared to the free Cy3 dye, as reported previously^7^. While the fluorescence intensity of free Cy3 decreased by approximately 15%, the conjugated NPs were barely affected by the continuous illumination. This indicates that the covalent conjugation protected the dye against oxidation and heat, which are associated with prolonged exposure to illumination^37,38^.

### Auxin loading within the proteinoid NPs

Auxins (Fig. S1a) are a class of plant hormones, which play a pivotal role in coordination of many behavioral and growth processes in the plant life cycle and are essential for the development of the plant body^27^. The auxin loading capability of the proteinoids prepared in the present work was analyzed by HPLC using calibration standard solutions of auxin, as described in the experimental part. Tryptophan monomeric units (Fig. S1b) are structurally similar to the molecular structure of auxin (Fig. S1a). Due to this similarity, proteinoid NPs containing tryptophan as part of the particle shell were chosen to entrap auxin, thereby improving its entrapment efficiency. Indeed, the results of this study indicate that most of the auxin – initially 5% by weight, relative to the proteinoids – was entrapped within P(EWH-PLLA) and P(KWH-PLLA) – 4.2 and 4.6%, respectively.

### Characterization of DA-Conjugated NPs

Dodecyl aldehyde (DA) – at 1 and 10 weight % relative to the proteinoid content – was covalently conjugated to the P(KEf) NPs by Schiff base bonds formed between the aldehyde groups of DA and primary amine groups on the NP surface^9^. The Schiff base bonds were reduced to stable C–N bonds by interaction with NaBH_3_CN, as described in Fig. 2^39,40^. Figure S3 illustrates that conjugation of DA to the P(KEf) NPs decreased their hydrodynamic diameter significantly from 284 ± 25 to 196 ± 20 and 112 ± 17 nm for 1% and 10% DA conjugation, respectively. On the other hand, our measurements indicated no significant difference in the dry diameter (76 ± 19 nm as shown in Table 3) of the pristine and the DA-conjugated P(KEf) NPs. The hydrodynamic size differences are probably due to hydrophobization of the NP surface as a consequence of the interaction with DA, which allows less water molecules to entrap and adsorb onto the NPs surface. Quantification of the DA content of the conjugated NPs via proton NMR and FTIR was not successful, probably due to the relatively low amount of bound DA, which could not be detected by these methods.

### Proteinoid NP stability

Instability of NPs is commonly a result of agglomeration^41^. The stability of the various non-modified and modified (Cy3 and DA-conjugated as well as auxin- containing) proteinoid NPs against agglomeration was investigated over time in two storage conditions – refrigerated aqueous continuous phase and freeze-drying. Aqueous dispersions of the proteinoid NPs (1 mg/mL) that were refrigerated at 4 °C for six months maintained the same size, as well as the original size distribution. The NPs were also freeze-dried to check the stability of the dried particles and redispersed at the original concentration^7^. This procedure did not affect sample sizes and size distributions, therefore both types of storage are suitable.

### XTT cell viability assay

**Figure 6:**
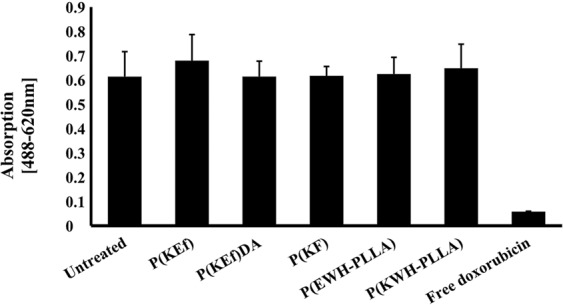
Cell viability levels of HUVEC after exposure to proteinoid NPs, measured by XTT assay. Cells (3 × 10^5^) were incubated for 48 h with proteinoid NPs dispersed in PBS (1 mg/mL) according to the experimental section. Untreated cells (positive control) were similarly incubated, as well as free doxorubicin, (100 nmol/ml, negative control). Each bar represents mean ± standard deviations of six separate samples.

An XTT cell viability assay was used to measure *in vitro* viability of human umbilical vein endothelial cells (HUVEC) following exposure to proteinoid NPs. HUVEC were exposed to proteinoid NPs that were dispersed in PBS (1 mg/mL) for 48 h. Control experiments were carried out – untreated HUVEC cells and free doxorubicin. Figure 6 exhibits the viability of the various cells: all proteinoid NPs sustained viability levels of 98–122%, indicating that the NPs themselves are non-toxic, therefore they can be used for various applications.

### Proteinoid NP phytotoxicity

**Figure 7:**
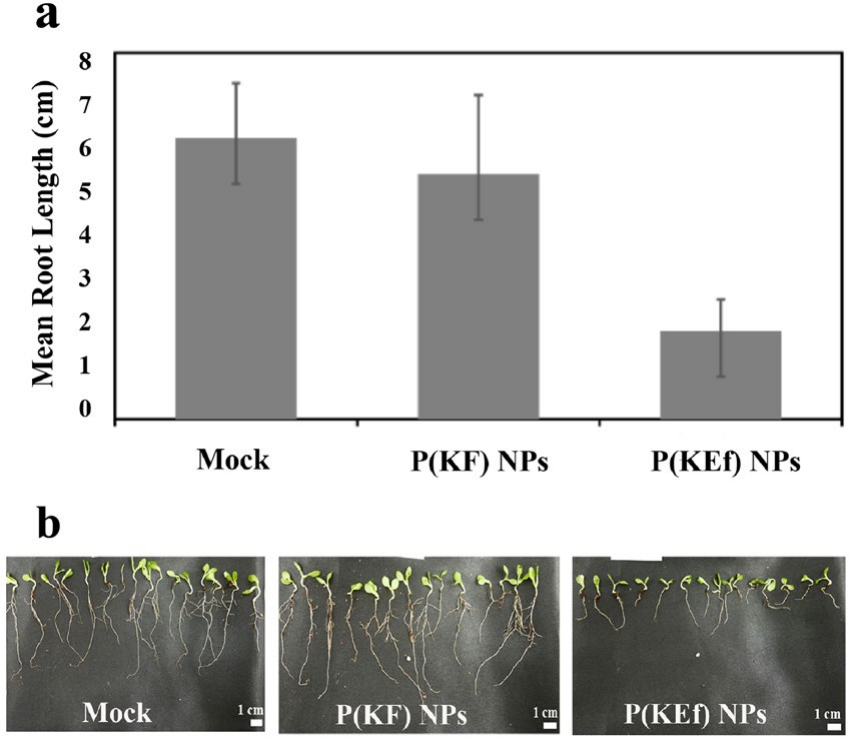
(**a**) Plant root measurements after treatment by water (mock), P(KF) NPs, and P(KEf) NPs after five days of growth in non-sterile media. Each bar represents mean ± standard deviations of six separate samples. **(b)** Germination of lettuce plants in absence (mock) and presence of P(KF) and P(KEf) NPs.

To evaluate the phytotoxic effects on germination, tests were first carried out with lettuce plants using P(KF) NPs. Figure 7a,b shows no significant difference in root length between plants treated by water (mock, control) and P(KF) NPs (6.1 ± 1.2 cm and 5.3 ± 1.7 cm) after five days of growth in non-sterile media. To assess the statistical significance the Turkey-Kramer method has been used, as described in the methodology part (2.9). This indicates that these proteinoid NPs are non-toxic, hence they are promising for delivery of agrochemicals in the field. Similar results were observed with P(EWH-PLLA) and P(KWH-PLLA) NPs. On the other hand, our study with P(KEf) NPs demonstrated statistically significant shorter roots length (1.9 ± 0.7 cm), indicating that they are toxic to the lettuce plants when tested in a germination assay, suggesting that Ef molecules (Fig. S1c) are released from the peptide chain in the course of biodegradation^42^ and act in similar fashion as free glufosinate, a widely used herbicide^43^. The Ef molecule is a well-known non-standard amino acid herbicide active only in the monomeric form^44^. Thus, Ef molecules are probably released from the peptide chain during biodegradation. In soil, glufosinate is readily degraded by microorganisms, with estimated half-lives between 1 and 25 days. Ef is strongly adsorbed on soil, mainly through its phosphonic acid group, therefore it is generally regarded as having low potential as a pre-emergent soil-applied herbicide. Its NP formulation, on the other hand, has such potential and may provide a new avenue for application of phosphate-containing herbicidal moieties.

#### Fluorescent imaging of proteinoid NP movement in plants

**Figure 8:**
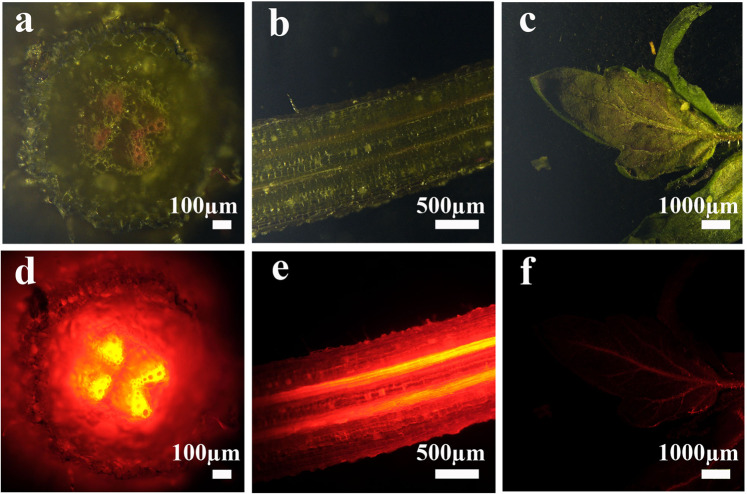
Nanoparticles applied to tomato roots. Herbicidal Cy3-conjugated P(KEf) NPs were added to liquid media; visible **(a–c)** and fluorescent **(d–f)** images taken after 24 h incubation show accumulation.

Using fluorescence stereomicroscopy, we visualized movement and accumulation of Cy3 conjugated NPs in differ plants organs. Accumulation of Cy3-conjugated P(KEf) NPs was observed in the roots of 14-day-old tomato plants after one day of incubation in the presence of the proteinoid NPs (Fig. 8). Based on the fluorescent images, the dye-conjugated P(KEf) NPs were found to penetrate roots and were closely associated with distinct root anatomical regions. Fluorescence-labeled NPs were detected in particular in the proto-xylem – primarily developing xylem sieves that consist of small, thin-walled cells. It is highly likely that the Cy3-conjugated NPs passed both the proto-xylem and the Casparian strip efficiently. The latter acts as a “second skin” in plant roots, providing cellular control that regulates loading of elements into plant vascular tissue. We could not identify labeling of meta-xylem – older xylem sieves with Cy3-conjugated P(KEf) NPs. This data demonstrates that the NPs were able to pass several plant tissue barriers and concentrated in the particular anatomical system. We then tested the distribution of the root-administered dye-conjugated NPs in leaves. Cy3-conjugated P(KEf) NPs were detected in leaves 24 h post application (Fig. 8c,f), indicating that the conjugated proteinoid NPs passed from the roots to leaves, demonstrating long-distance movement of NPs in plants.

**Figure 9:**
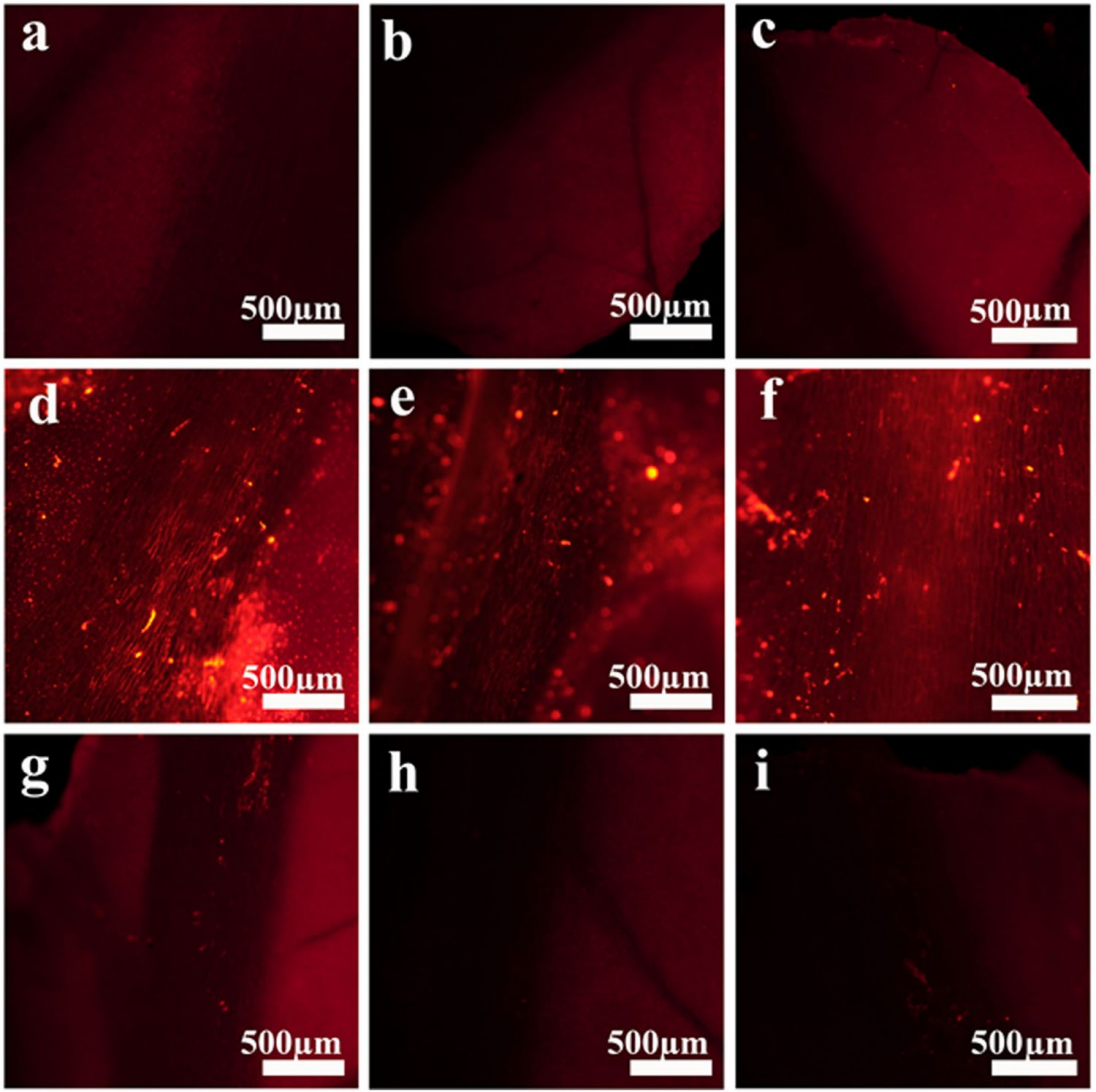
NPs applied to the leaves translocate to other leaves: herbicidal Cy3-conjugated P(KEf) NPs were administered foliarly to lettuce plants. Pieces of treated and untreated leaves imaged after a 72 h incubation period using a fluorescent binocular showed gradual accumulation in the vascular plants system – **(a–c)** untreated plants, **(d–f)** leaves treated with dye-conjugated P(KEf) NPs, **(g–i)** untreated leaves.

We then examined the NP movement via fluorescent imaging and tested the distribution of the foliarly administered P(KEf) NPs to other plant organs using lettuce plants as a model. NPs were applied to a single leaf, and limited movement was observed to other leaves (Fig. 9). A low fluorescent signal was detected in untreated leaves after 72 h. As in tomato roots, the low signal was located mainly in the vascular region of the leaves, indicating movement of NPs via the xylem network. These observations indicate that the proteinoid NPs translocated to some extent to other leaves.

#### Targeted agrochemical delivery using proteinoid NPs

Previous work suggested that Au nanoparticles could deliver phytohormones into plant tissue culture (*ex vivo*)^45^. The plant hormone auxin (Fig. S1a) is essential for plant growth^27^, and has been implicated in numerous aspects of plant physiology – from germination to senescence; at a molecular level it serves as a rheostat for both positive and negative transcriptional regulation of target genes^46^. The accumulation of NPs in plants suggests the possibility of agrochemical delivery using the auxin-encapsulated NPs. For this purpose, we used DR5:VENUS transgenic background tomatoes combining the auxin-responsive promoter DR5 with yellow fluorescent protein (YFP) expression, which indirectly reflect auxin concentration. Exogenously applied auxin causes over-expression of YFP protein in DR5 plants, and thereby allows quantification of hormones in living tissue^27,47^.

**Figure 10:**
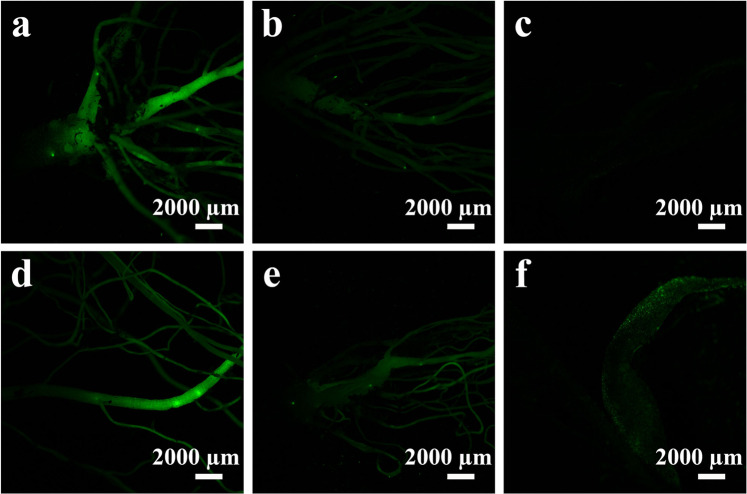
Tomato DR5:YFP plant activation by pristine and auxin-encapsulating P(KWH-PLLA) and P(EWH-PLLA) NPs. One-month old DR5 tomato seedlings were placed in soil in pairs. Each pair was treated with 350 µL aqueous dispersion of pristine (**b** and **e**) and auxin-encapsulating proteinoid NPs (10 mg/mL, **a**,**c** and **d**,**f)**. Plants were grown under light in a growth room for two days, and fluorescence of leaves (**c** and **f**) and roots (**a**,**b**,**d** and **e**) was observed with a YFP filter.

Treatment with auxin-encapsulating P(KWH-PLLA) and P(EWH-PLLA) NPs caused activation of DR5:YFP expression in lateral roots, as shown in Fig. 10 (pictures **a** and **d**, respectively). On the other hand, expression in leaves of tomato plants was not significantly detected by the auxin-encapsulating P(KWH-PLLA) and P(EWH-PLLA) NPs, as illustrated in pictures **c** and **f**, respectively, or by the non-ecapsulating (pristine) proteinoid NPs. Roots of tomato plants were not clearly detected by pristine P(KWH-PLLA) and P(EWH-PLLA) NPs, as shown in pictures **b** and **e**, respectively. Taken together, these observations indicate that the proteinoid NPs accumulated mainly through roots, with subsequent release of the phytohormone in the nucleus or in the cytoplasm.

#### Surface modification for improvement of foliar application of herbicidal proteinoids

Today, many of the most successful herbicides are amino acids analogues. Ef (Fig. S1c) – a naturally found non-protein amino acid analogue of glutamate – is a specific inhibitor of glutamine synthetase^48^. Ef is produced from an inactive protoxin tripeptide^49^. Gp (glyphosate, Fig. S1d) – the most successful herbicide, structurally related to glycine – is a specific inhibitor of 5-enolpyruvyl-shikimate synthase (EPSPS), an enzyme involved in aromatic amino acids biosynthesis.

**Figure 11:**
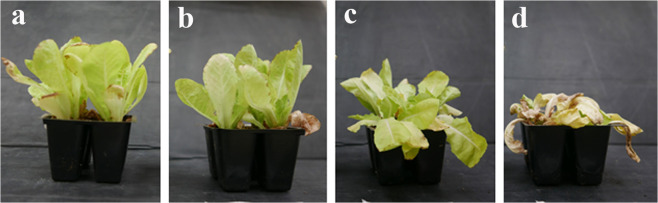
Increased herbicidal activity of the P(KEf) NPs obtained by surface conjugation with increasing concentrations of DA. Tests were performed with leaves of one week-old lettuce plants, treated once with proteinoid NPs: **(a)** untreated, **(b)** P(KEf) NPs, **(c)** 1% DA-conjugated NPs and **(d)** 10% DA-conjugated proteinoid NPs.

Environmental and health issues associated with these herbicides have become an increasingly major concern due to potential toxicity to humans and animals^50–53^ Therefore, we decided to construct controllable proteinoid NPs with a commercial herbicidal amino acid, employing P(KEf) NPs as a model. In order to improve foliar uptake given the hydrophobic nature of the leaf surface, we modified the NP surface with different concentrations of the hydrophobic surfactant dodecyl aldehyde (DA), as shown in Fig. 2. Treatment of DA with P(KEf) NPs indeed illustrated the possibility of achieving a phytotoxic effect of herbicidal NPs via foliar application, although the non-modified aforementioned NPs are in general weaker herbicides compared to commercial products. Surface modification with DA drastically improved the phytotoxic effect of the P(KEf) NPs, opening the way to delivery of agrochemicals to plants via foliar application (Fig. 11).

According to the results, the phytoxic effect of these NPs increased remarkably with higher concentrations of bound DA. It is well established that efficacy of agrochemicals is enhanced by increasing foliar uptake via formulation with surfactants^54^. In addition, based on our previous observation, proteinoid NPs of commercial herbicides are more stable. Application of Ef usually induces in the first stage visible signs of necrosis and brown spots. To co-localize Ef-containing NPs, we covalently attached a Cy3 fluorescent marker to the P(KEf) NPs. Using such fluorescent NPs, we could easily localize necrotic regions. Localization of Cy3 fluorescence in the necrotic core of the leaf was confirmed by confocal binocular. As shown in Fig. S4, the conjugated NPs exhibited strong accumulation in the necrotic areas of leaves, indicating co-localization of fluorescent signal and toxic effect of glufosinate released by the NPs.

Figure 11 illustrates that P(KEf) NPs alone have small damaging effect on the lettuce (Fig. 11b) probably due to low permeability of the hydrophilic proteinoid NPs through the hydrophobic leaf. On the other hand, conjugation of DA leads to increased damage due to improved permeability of these hydrophobic NPs through the leaf. Furtheremore, P(KEf) NPs reacted with 10% DA lead to total death (Fig. 11d) while those treated with 1% DA showed much less damage (Fig. 11c).

## Summary and conclusions

Functional polymeric NPs present a rapidly growing field of biotechnology. In medicine, NPs are used for imaging, drug delivery, controlled release, cell labeling and separation, and therapy and diagnosis of cancer and neurodegenerative disorders^55^. Here, we aimed to explore the use of functional NPs for agricultural purposes. Thereby, we prepared and characterized four model proteinoid polymers – P(KEf), P(KF), P(EWH-PLLA) and P(KWH-PLLA) – by bulk thermal step-growth polymerization of the appropriate amino acid monomers. Proteinoid hollow NPs (nanocapsules) were then formed by a self-assembly process of the former proteinoid polymers. The agricultural activity of these NPs may be controlled by the composition of the polymeric shell of these NPs, by entrapment of desired molecules during the self-assembly process, or by surface modification. Using microscopic techniques, we were able to localize the fluorescent-labeled NPs and observe the accumulation of NPs in the plant’s vascular system. In addition, using genetically modified plants that express fluorescent protein and respond to the level of the hormone auxin, we demonstrated, using the same microscopic techniques, the possible delivery of encapsulated agrochemicals into cells.

We chose the herbicidal non-standard amino acid Ef as a model for introduction to the proteinoid NP shell. P(KF), P(EWH-PLLA) and P(KWH-PLLA) NPs seem to be non-toxic to endothelial cells of the human umbilical vein (HUVEC) and lettuce plants. Nevertheless, P(KEf) NPs were found to be toxic to lettuce plants. The Ef molecule is a well-known non-standard amino acid herbicide active only in the monomeric form^44^. Thus, our work suggests that Ef molecules are probably released from the peptide chain through biodegradation.

Altogether, this work illustrated the delivery of several types of agrochemicals (herbicides and hormones) using the proteinoid NP platform in plants. In future work, the present study should be extended to additional amino acids (e.g., proline, cysteine, alanine and arginine), potential growth enhancers (e.g., metal ions such as Zn, Fe and Mn) and herbicides such as glyphosate. Special care should be taken to control and study the rate of NP degradation and subsequent payload release by adding other functional groups to the polypeptide chains. For example, changing the content and/or molecular weight of PLLA molecules in the polymerization process may significantly affect the degradation rate of the NPs. Longer periods of controlled release may alternatively be achieved by coating the proteinoid NPs with PEG of different length by means of coupling PEG-NHS to primary amine groups on the proteinoid surface^9^.

The delivery of active compounds into plants presents another step in the expansion of nanotechnology to this important field, and promises higher yields, as well as better methodologies for agricultural research^56^. Our results demonstrate efficient preparation and delivery of agrochemicals to plants in a stable and controlled fashion.

## Supplementary information

Supplementary information
