# Supplementary material for: Engineering and use of proteinoid polymers and nanocapsules containing agrochemicals
Source: Sci Rep. 2020 Jun 8;10:9171. doi: 10.1038/s41598-020-66172-w (PMC7280236; doi:10.1038/s41598-020-66172-w)
Supplement: Supplementary file 1 — Supplementary information [file 41598_2020_66172_MOESM1_ESM.docx]

**Engineering and use of proteinoid polymers and nanocapsules containing agrochemicals**

**Elisheva Sasson^1^, Ruth Van Oss Pinhasi^2^, Shlomo Margel^1^* and Liron Klipcan^2^***

^1^ The Institute of Nanotechnology and Advanced Materials, Department of Chemistry, Bar-Ilan University, Ramat-Gan, Israel 5290002

^2^ Gilat Research Center, Agricultural Research Organization, Mobile Post Negev 2, Israel 8531100

*Corresponding authors

E-mail addresses:

elisheva.sa@gmail.com (E. Sasson), ruthvop@volcani.agri.gov.il (R. Van Oss Pinhasi), shlomo.margel@biu.ac.il (S. Margel), lironk@volcani.agri.gov.il (L. Klipcan)

**Supporting Information**

**Figures S1–S4**

Figure S1. Structures of active molecules discussed in the manuscript: (a) auxin, (b) tryptophan, (c) glufosinate, and (d) glyphosate.

**
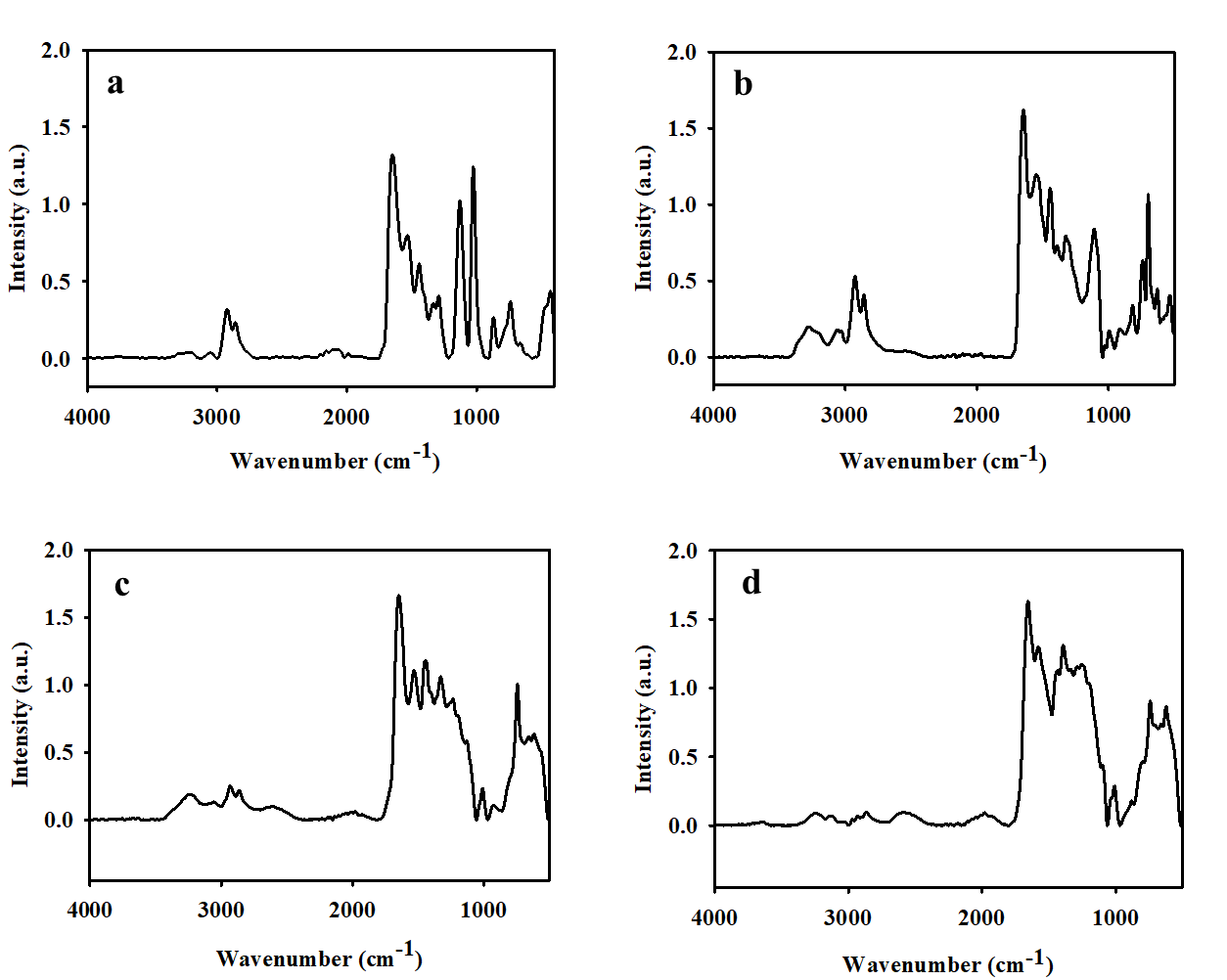
**

Figure S2. FTIR spectra of : (a) P(KEf), (b) P(KF), (c) P(KWH-PLLA), and (d) P(EWH-PLLA) NPs.

**

**

Figure S3. DLS histograms of pristine (solid line) and DA-conjugated (dashed line – 1%, dotted line – 10%) P(KEf) NPs.

**
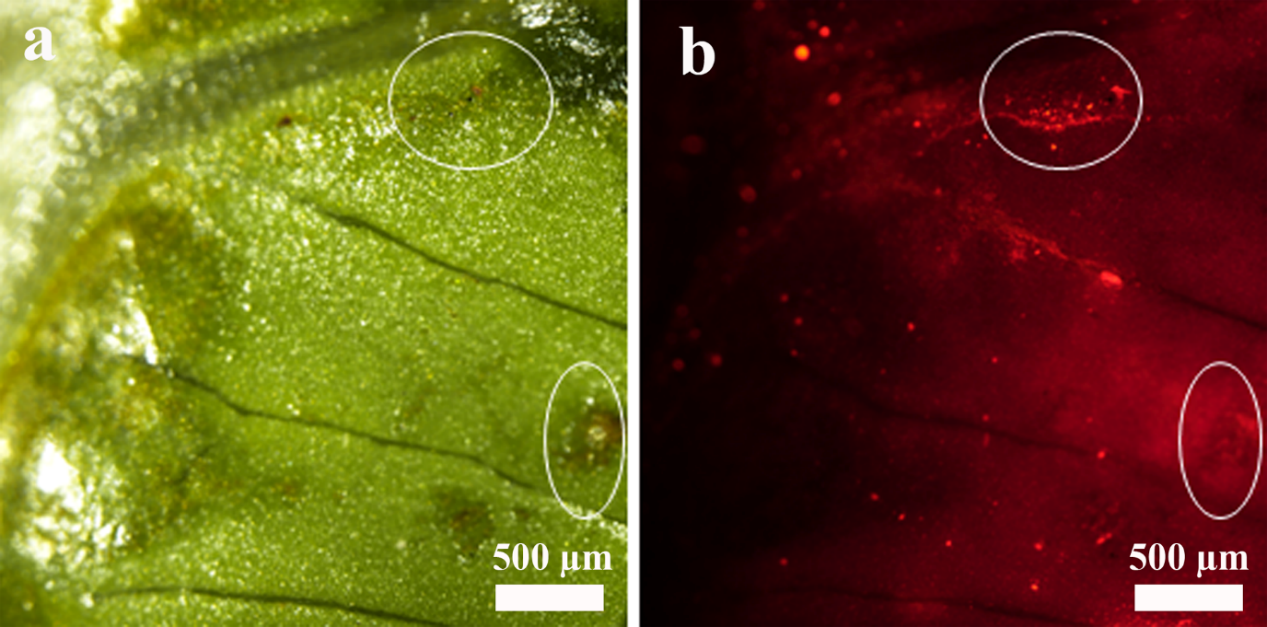
**

Figure S4. Confocal microscopic images showing localization of Cy3-conjugated P(KEf) NPs in lettuce leaf: (a) vis. *vs.* (b) fluorescence. Circles indicate brown spots.
